# Human Pluripotent Stem Cells: A Unique Tool for Toxicity Testing in Pancreatic Progenitor and Endocrine Cells

**DOI:** 10.3389/fendo.2020.604998

**Published:** 2021-01-19

**Authors:** Erin M. MacFarlane, Jennifer E. Bruin

**Affiliations:** Department of Biology & Institute of Biochemistry, Carleton University, Ottawa, ON, Canada

**Keywords:** stem cells, diabetes, pollution, beta cells, pancreas development, toxicology

## Abstract

Diabetes prevalence is increasing worldwide, and epidemiological studies report an association between diabetes incidence and environmental pollutant exposure. There are >84,000 chemicals in commerce, many of which are released into the environment without a clear understanding of potential adverse health consequences. While *in vivo* rodent studies remain an important tool for testing chemical toxicity systemically, we urgently need high-throughput screening platforms in biologically relevant models to efficiently prioritize chemicals for in depth toxicity analysis. Given the increasing global burden of obesity and diabetes, identifying chemicals that disrupt metabolism should be a high priority. Pancreatic endocrine cells are key regulators of systemic metabolism, yet often overlooked as a target tissue in toxicology studies. Immortalized β-cell lines and primary human, porcine, and rodent islets are widely used for studying the endocrine pancreas *in vitro*, but each have important limitations in terms of scalability, lifespan, and/or biological relevance. Human pluripotent stem cell (hPSC) culture is a powerful tool for *in vitro* toxicity testing that addresses many of the limitations with other β-cell models. Current *in vitro* differentiation protocols can efficiently generate glucose-responsive insulin-secreting β-like cells that are not fully mature, but still valuable for high-throughput toxicity screening *in vitro*. Furthermore, hPSCs can be applied as a model of developing pancreatic endocrine cells to screen for chemicals that influence endocrine cell formation during critical windows of differentiation. Given their versatility, we recommend using hPSCs to identify potential β-cell toxins, which can then be prioritized as chemicals of concern for metabolic disruption.

## Introduction

### Diabetes Pathogenesis

Diabetes is a chronic disease characterized by high blood sugar levels and devastating secondary health complications (1). In 2019, there were >460 million people with diabetes worldwide, which translates to roughly 1 in 11 adults ages 20-79 years. For those over the age of 65, diabetes rates further increase to 1 in 5 (1). Moreover, the International Diabetes Federation projects that diabetes incidence will increase by 51% over the next 25 years to exceed 700 million adults worldwide.

Glucose homeostasis is maintained by the exquisite balance of hormones secreted from pancreatic islets. The predominant islet cell type is the β-cell, which secretes insulin in a tightly regulated manner in response to glucose and other stimuli (2, 3). Type 1 diabetes (T1D), accounting for ~10% of patients with diabetes, is caused by autoimmune destruction of β-cells leading to insufficient insulin production. Type 2 diabetes (T2D) accounts for ~90% of cases and was classically thought of as a disease of insulin resistance. However, we now appreciate that β-cell dysfunction and loss of β-cell mass are also central to T2D pathogenesis (4–6). The critical role of β-cells in driving diabetes risk is further confirmed by genome-wide association studies, which find that most loci influencing T2D risk are involved in regulating insulin secretion (7–9). These studies also emphasize that rising diabetes rates cannot simply be explained by genetics, but rather must be influenced by environmental factors (9, 10). For example, there is strong epidemiological evidence linking exposure to persistent organic pollutants (POPs) with increased T2D incidence (11–29) and β-cell dysfunction (28–30) in humans. However, basic research in clinically relevant models is needed to understand the potential causal role for environmental contaminants in diabetes pathogenesis and to explore underlying tissue- and cell-specific mechanisms of toxicity.

### Environmental Contaminants

Environmental pollutants are a major global concern due to their wide-ranging acute and chronic adverse effects on human health (31). With over 84,000 chemicals in commerce, there is an urgent need to develop tools for extensive chemical screening and toxicity testing (32). Environmental contaminants fall within a wide range of classes, including but not limited to POPs (e.g., pesticides, polychlorinated biphenyls (PCBs), dioxin-like compounds), estrogen analogues (e.g., bisphenol A (BPA), used in polycarbonate plastics), phthalates (used in cosmetics, paints, textiles), heavy metals, perfluorinated chemicals (e.g., perfluorooctane sulfonate (PFOS) used in food packaging and fire-fighting foams), and flame retardants (e.g., polybrominated diphenyl ethers, organohalogen compounds, organophosphates esters) (33–35). Contaminants can be further classified by their mechanism of action. For example, chemicals that impair proper hormone function are referred to as endocrine-disrupting chemicals (EDCs) (33, 36–40) and those that disrupt metabolism are classified as metabolism-disrupting chemicals (MDCs) (41–43). Despite restrictions on many environmental pollutants, these chemicals continue to persist in the environment, contaminating food and water sources, and remain detectable in human tissues (44, 45).

Biomonitoring is essential for tracking human contaminant exposure and predicting adverse health outcomes (46, 47). However, this is a reactive approach to evaluating the impact of toxins on human health. Ideally, we need to efficiently screen chemicals for toxicity in relevant model systems prior to their release into the environment. Since pollutants often accumulate in tissues, effectively creating a chemical mixture cocktail (48), we also need to consider the combined effects of complex chemical mixtures. Dose and duration of exposure add additional layers of complexity. For example, POPs have long half-lives of years to decades (49), but the shorter lifespan of other chemicals such as BPA and phthalates is also not trivial. Much like hormones, EDCs can exert their effects on the human body at low concentrations over an extended period of time (50). Despite being excreted within days, frequent consumption of these pollutants results in chronic, low dose exposure over time (51, 52). Furthermore, nonlinear dose-responses are frequently seen with EDCs, so acute high dose studies may not accurately predict adverse health outcomes of chronic or subacute low dose exposures (42, 53, 54). The need to consider chemical exposures ranging from acute high doses to chronic low doses, as well as individual chemicals and complex mixtures, further emphasizes the importance of scaling up toxicity testing capacity.

### Developmental Origins of Disease

Another important consideration for toxicology studies is the timing of exposure to environmental contaminants. Gestational or early life stressors, such as undernutrition or overnutrition, are linked to a variety of adult-onset diseases - termed developmental origins of health and disease (DOHaD) (55, 56). For instance, low birth weight and early life “catch up” growth are well-established risk factors for developing metabolic disease later in life (57, 58). Maternal-fetal exposure to POPs has been linked to adverse outcomes such as reduced birth weight, disruption of hormone levels in cord blood, and changes in epigenetic markers of development (59–61). There is also mounting epidemiological evidence suggesting a possible link between early-life environmental contaminant exposure and long-term metabolic dysfunction (62–66). More epidemiology is needed and important cohort studies like the Maternal-Infant Research on Environmental Chemicals (MIREC) continue to track long-term metabolic outcomes in offspring (67–70). However, it takes decades to truly establish a link between early-life exposure and long-term adverse health outcomes. *In vitro* model systems that allow for toxicity screening in developing human cells will be a powerful starting point for studying DOHaD.

### Perspective Overview

**Figure 1:**
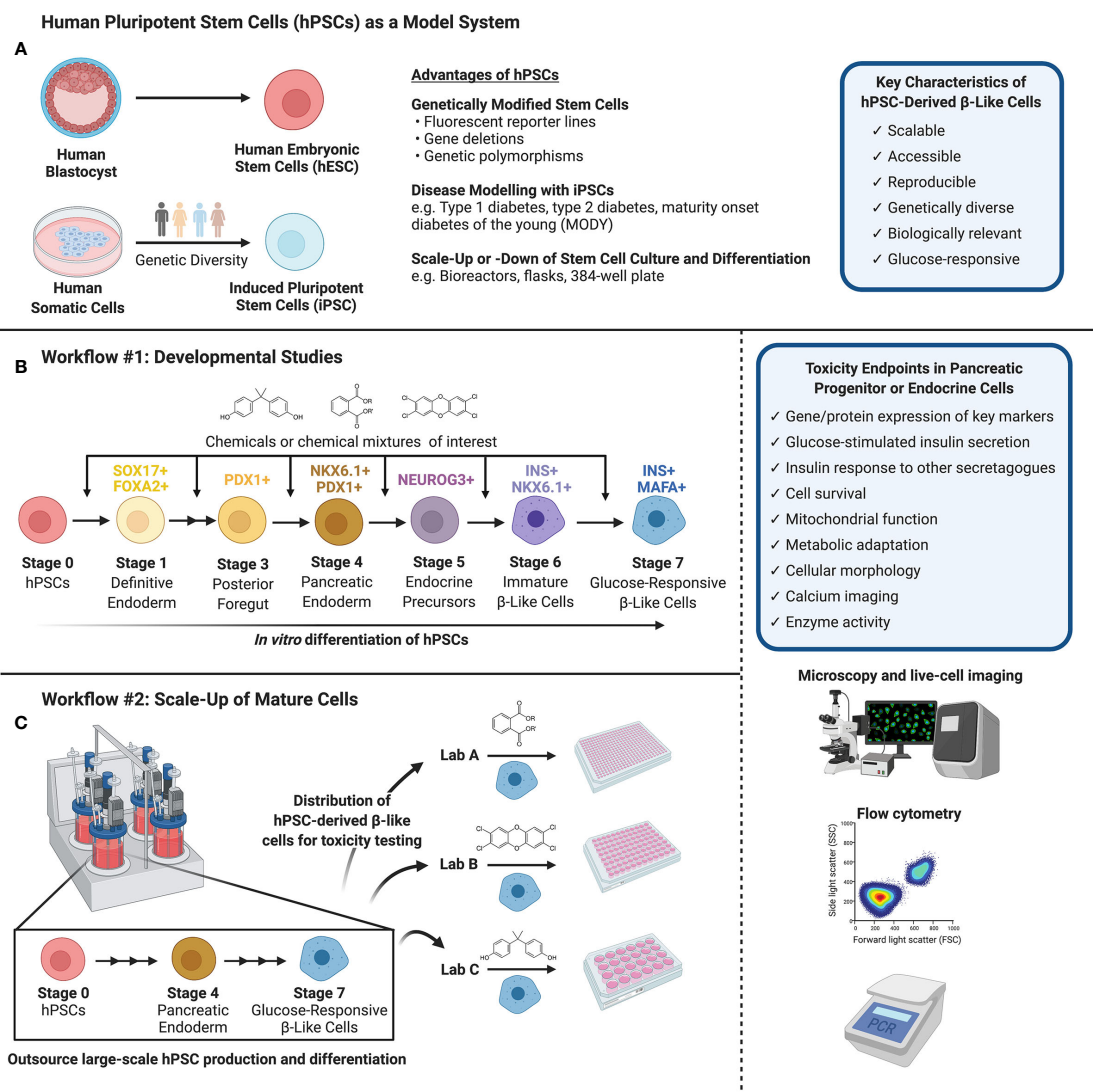
**(A)** Human pluripotent stem cells (hPSCs) can be isolated from the inner cell mass of a human blastocyst (human embryonic stem cells, hESCs) or obtained *via* reprogramming of human somatic cells obtained from genetically diverse donors (induced pluripotent stem cells, iPSCs). hPSCs are versatile in their capacity for genetic modifications and disease modeling and may be scaled up or down to suit a variety of experimental conditions. **(B)** Workflow #1 illustrates how hPSCs may be used to screen chemicals or chemical mixtures of interest throughout pancreas development. hPSCs can be differentiated into pancreatic endoderm using published protocols or commercially available differentiation kits, and further into maturing, glucose-responsive β-like cells. Chemicals can be introduced at different days or stages of differentiation to mimic environmental exposures at different windows of pancreas development. **(C)** Workflow #2 demonstrates the capacity to outsource hPSC expansion and large-scale differentiation, allowing individual labs to conduct toxicity screening of specific chemicals/chemical mixtures using glucose-responsive β-like cells generated in a central location. **(B, C)** We suggest a number of potential toxicity endpoints, such as cell survival, insulin secretion, and mitochondrial function. Common analytical methods include but are not limited to microscopy and live-cell imaging, flow cytometry to quantify cell populations throughout differentiation, and PCR to assess gene expression (Created with BioRender.com).

There is an urgent need to identify environmental contaminants, specifically EDCs or MDCs, that contribute to diabetes pathogenesis. To do so, we must consider non-classical toxicological endpoints in a wide variety of tissues involved in regulating metabolic homeostasis. This means thinking beyond typical hepatoxicity endpoints and considering diverse metabolic targets such as neuroendocrine cells, enteroendocrine cells, white or brown adipocytes, skeletal muscle, thyroid gland, and pancreatic endocrine cells (38, 40, 71). While injury to any of these tissues would potentially disrupt energy homeostasis, we propose that pancreatic endocrine cells should be a high priority for toxicity testing to identify MDCs of concern for diabetes pathogenesis. In this Perspective Article, we discuss a range of endpoints that could be considered in the context of β-cell toxicity. We also discuss various model systems available for toxicity testing, including the numerous advantages of human pluripotent stem cells (hPSCs). In particular, we propose hPSCs as a unique model system for evaluating toxicity both during critical windows of β-cell development and in glucose-responsive adult β-like cells (**Figure 1**).

## Toxicity Testing in Pancreatic β-Cells

Despite mounting evidence implicating pollutants as metabolic disruptors, the pancreas has not been extensively studied in the toxicology field (40, 42). Interestingly, the occasional biodistribution studies that include pancreas tissue report a slower elimination of lipophilic pollutants in the pancreas compared to liver or adipose (72, 73). Xenobiotic metabolism enzymes, such as cytochrome P450 (Cyp) enzymes, are useful biomarkers for direct cellular exposure to pollutants. We have reported induction of *Cyp1a1* in mouse and human islets following direct exposure to TCDD/dioxin or dioxin-like pollutants *in vitro* and in mouse islets following systemic administration of TCDD *in vivo (* 73). Moreover, in pregnant TCDD-exposed mice, *Cyp1a1* was induced 17-fold in pancreas compared to only 3-fold and 7-fold in liver and adipose, respectively (74). Therefore, pancreatic cells are not only directly exposed to pollutants *in vivo*, but may even act as a “sink” for long-term storage of lipophilic chemicals, similar to adipose depots.

There is mounting evidence that a wide range of environmental contaminants can directly impact β-cell function. For example, BPA, a non-persistent additive commonly used in plastic products, acutely increases insulin secretion in mouse and human islets *via* inhibition of K_ATP_ channels and increased Ca^2+^ signaling (75), whereas longer-term BPA exposure inhibits Ca^2+^ entry and reduces insulin secretion (76). Newer BPA-replacement chemicals, BPS and BPF, also disrupt mouse β-cell function (77). Exposure to POPs, including organochlorine pesticides and a PCB mixture, directly inhibited insulin secretion in a rat β-cell line (INS-1E cells) (30). A “northern contaminant mixture”, containing 20 different POPs at environmentally relevant concentrations, also suppressed insulin secretion in rats *in vivo* and in a rodent β-cell line (MIN6 cells) *in vitro (* 78). Additionally, we and others have shown that dioxin suppresses insulin secretion in rodent islets (73, 79, 80) and human islets (73). Interestingly, acute high-dose dioxin exposure caused β-cell apoptosis in male mice but not female mice (81), whereas prolonged low-dose dioxin exposure led to impaired β-cell adaptation to high fat diet feeding in female but not male mice (74, 82).

Given the critical role for β-cells in diabetes pathogenesis, environmental toxins that adversely impact β-cells are likely to disrupt overall glucose homeostasis or at minimum, increase diabetes risk. Importantly, there are numerous plausible avenues for toxin-induced β-cell injury that could lead to adverse metabolic health outcomes. Below, we propose key toxicity endpoints for β-cells. This is not intended to be a comprehensive list of potential adverse outcomes, but rather examples that should be considered as a starting point for identifying MDCs that act as β-cell toxins.

### Insulin Secretory Defects

Pancreatic β-cells are highly specialized to synthesize, process, store, and secrete insulin rapidly and sustainably in response to numerous physiological stimuli, including glucose (2). There is a rapid first phase of insulin secretion within minutes of a glucose stimulus, followed by a sustained second phase that can last for several hours. Glucose-stimulated insulin secretion is amplified by other nutrients, such as fatty acids and amino acids, and binding of gut-derived hormones (GLP-1, GIP) to incretin receptors. Dysregulated glucose-induced insulin secretion is a well-established feature of T2D (83–87) and also reported in T1D patients prior to disease onset (88–92), suggesting a possible link between insulin secretory defects and activation of inappropriate autoimmune responses. Therefore, environmental contaminants that interfere with any aspect of the complex insulin secretory pathway (e.g., glucose sensing, mitochondrial metabolism, ion flux, exocytotic machinery, sensitivity to amplification signals) could adversely affect the fine-tuned ability of β-cells to couple insulin secretion with a nutrient secretagogue. Furthermore, defects in the timing of insulin release, either the rapid first-phase or the sustained second-phase response, could impact overall glycemic control. Importantly, β-cell dysfunction is not just insufficient or decreased insulin secretion, but also refers to overproduction of insulin. Hyperinsulinemia is not only an adaptation to insulin resistance but can also be the primary defect that drives obesity and insulin resistance (93–96). Therefore, toxins that increase insulin secretion – either inappropriate insulin release under basal glucose conditions or excessive insulin secretion following a nutrient stimulus – should also be considered potential MDCs.

### Loss of β-Cell Mass

Patients with T1D display near complete loss of β-cell mass (> 80% reduction) at the time of disease onset (6). Although less extreme, individuals with T2D also have reduced β-cell mass (5, 6, 97, 98), which may be caused by a combination of increased β-cell death, insufficient β-cell proliferation, or impaired β-cell neogenesis. β-cell mass is generally increased in overweight or obese non-diabetic subjects compared to lean controls but reduced by 24%–65% in patients with T2D (5, 6, 99). Loss of β-cell mass can be detected by measuring the α-cell to β-cell ratio, which is consistently higher in patients with T2D (97). Therefore, environmental toxins that disrupt β-cell mass, for example by inducing β-cell apoptosis or preventing β-cell expansion, should be flagged as potential MDCs.

### Impaired β-Cell Adaptation

Healthy β-cells have fine-tuned mechanisms for adapting to fluctuations in energy supply and insulin demand to maintain appropriate glucose homeostasis (100). Examples of complex compensatory mechanisms include a) regulation of key β-cell transcription factors that control the β-cell transcriptome, b) altered activity or expression of metabolic enzymes to allow for flexibility in nutrient metabolism, and c) increased β-cell proliferation to expand functional mass of β-cells. Failed compensatory insulin secretion and expansion of β-cell mass during insulin resistance are important predictors of diabetes susceptibility (99, 101, 102). Therefore, toxicology studies should consider the ability of β-cells to appropriately compensate under conditions of fasting or insulin resistance. If only direct cellular toxicity is tested without considering interactions with other metabolic challenges, potential MDCs will be overlooked.

### Impaired β-Cell Development

The number of pancreatic progenitors present throughout embryonic development is a critical determinant of β-cell mass and pancreas size in adulthood, unlike the closely related endoderm-derived liver which can fully compensate following partial progenitor cell ablation (103). Therefore, an infant born with reduced β-cell mass may have a compromised ability to adapt to metabolic stressors later in life. Additionally, overproduction of insulin at birth caused by inappropriate β-cell expansion or accelerated maturation could lead to the development of insulin resistance and obesity (as described above) *(* 94–96, 104). Therefore, a starting point for predicting long-term adverse metabolic health outcomes following intrauterine chemical exposure would be screening for chemicals that alter β-cell development. We propose that “developmental MDCs” could be prioritized, in part, based on whether they influence the formation of β-cells – either decreasing or increasing numbers – during critical windows of development.

## Traditional Models for Toxicity Testing in Pancreatic Endocrine Cells

### *In Vivo* Rodent Models

*In vivo* rodent models are an important tool for toxicity testing, but pose a significant technical barrier to high throughput screening (105) and are limited in their ability to predict human outcome. In a largescale study of pharmaceutical toxicity testing, rodents were predictive of human toxicity for only 43% of tested compounds, and demonstrated poor concordance for liver and endocrine toxicity (106). Further, human populations are genetically diverse and exist amongst variable exogenous factors, whereas laboratory animals are genetically uniform and housed within controlled environments to support reproducibility. While *in vivo* testing is necessary for assessing the impact of chemicals on a whole organism rather than just a single cell or tissue type in isolation, there has been a shift towards first prioritizing chemicals using *in vitro* model systems with higher throughput capacity (107, 108).

### Immortalized β-Cells

#### Rodent Cell Lines

Immortalized rodent β-cell lines are robust in culture and highly proliferative, making them a useful tool for large-scale studies. Unfortunately, their replication capacity limits their applicability as a model for human β-cells, which have minimal ability to proliferate (99, 109). In addition, commonly used insulin-secreting rodent β-cell lines, including β-TC-6 (mouse), MIN6 (mouse), and INS-1 (rat) cells, have varying degrees of glucose-responsiveness (110, 111). Immortalized cell lines also tend to be genetically unstable in culture for extended periods of time. Clonal INS-1E cells have higher stability than the INS-1 parental line and maintain their insulin content in passages >40 (112), but both INS-1/INS-1E cells are cultured with the toxic reducing agent, 2-mercaptoethanol, which further limits their biological relevance for toxicity testing.

Despite these limitations, INS-1 823/13 cells were comprehensively evaluated as a potential pollutant-screening system. This cell line was found to be adequately glucose-responsive, but the insulin secretory responses to key control compounds and pollutants deviated substantially from previous reports (113). The authors concluded that INS-1 823/13 cells were lacking key β-cell characteristics and deemed inadequate as a diabetogenic pollutant screening system (113). We reached a similar conclusion after testing immortalized pancreatic endocrine cells for their response to TCDD/dioxin, a POP that acts *via* the aryl hydrocarbon receptor (AhR). In primary mouse and human islets, TCDD significantly upregulated CYP1A1 gene expression and enzyme activity, whereas immortalized β-cell (INS-1, MIN6, β-TC6) and α-cell (α-TC1 and α-TC3) lines showed no evidence of AhR activation by TCDD (73). Our findings confirm that important discrepancies exist in the cellular machinery between primary and immortalized cell lines.

#### Human Cell Lines

Novel engineered human β-cell lines, EndoC-βH1 and EndoC-βH2, are a useful tool for studying β-cell physiology and drug responses (114, 115). EndoC-βH1 cells were engineered from human fetal pancreatic buds transduced with an SV40LT-expressing lentiviral vector under the control of an insulin promoter. These cell lines are glucose-responsive *in vitro* and have minimal expression of other pancreatic genes (114–116). Their main drawback is the limited capacity for expansion *in vitro*, which is more biologically appropriate than rodent β-cell lines, but less practical. With a doubling time of approximately 7 days, their potential for high-throughput toxicity studies is limited. While proliferation can be stimulated with SerpinA6, STC1, and APOH (114), using compounds that alter cellular physiology is not ideal for toxicity testing. Additionally, as EndoC β-cells are a product of oncogenesis, there is undoubtedly alterations to normal pathways (117).

### Primary Isolated Islets

#### Non-Human Islets

Primary rodent islets are more biologically relevant than immortalized rodent β-cell lines, but there are important distinctions between human and rodent islets that must be considered. For example, the distribution and composition of endocrine cells, vasculature, innervation, proliferation, and insulin secretion all differ between human and rodent islets (118, 119). Pig islets are more similar to human islets (120). Interestingly, islets isolated from juvenile porcine pancreata have greater expression of β-cell-related genes compared to those isolated from adult pigs (121), but are functionally immature and require *in vitro* maturation following isolation (122–124). Further, it is possible to isolate up to 5,000 islets/g juvenile porcine pancreas (122), compared to a typical yield of ~200–400 islets total per mouse pancreas and 600–800 islets per rat pancreas depending on the strain (125–127). Thus, pig islets may be a useful tool for toxicity screening, although species differences will always remain a concern for translation.

#### Human Islets

Human islets are currently the gold standard for a physiologically relevant model to study the endocrine pancreas *in vitro* due to their cellular composition, human origin, and genetic diversity. Human islets are harvested from deceased organ donors and great strides have been made to ensure that high quality donor islets are broadly available for research (128, 129). However, even with the highest quality isolation procedures, the pancreas begins to autodigest after death, resulting in decreased cell viability and sample quality (119). Human islets have a limited functional *in vitro* lifespan with current tissue culture protocols, although advances in the field are ongoing. For example, islets cultured on specific matrices maintain glucose-stimulated insulin secretion for at least 7 days in culture (130). The number of purified islets per donor also varies; while an average healthy adult pancreas houses over 3 million islets (118), between 200,000 and 500,000 islets typically remain post-purification (131). This, coupled with the limited proliferation of human β-cells, presents a critical barrier to scalability and longevity for toxicology testing. Therefore, human islets are an excellent resource for *in vitro* chemical testing at a smaller scale, wherein endpoints such as glucose-stimulated insulin secretion, islet morphology, mitochondrial function, and gene expression can be assessed in biologically diverse organ donors. Numerous factors, such as donor sex, age, and body mass index, will influence islet function *ex vivo* and thus impact biological reproducibility. However, the genetic and environmental diversity of human organ donors (132, 133) also offer a unique opportunity for toxicity testing.

## Stem Cells are a Unique Tool for Pancreas Toxicology Studies

HPSC culture offers a unique *in vitro* solution to address the need for high-throughput screening of environmental toxins in a variety of biologically relevant mature cell types, as well as in differentiating or “developing” immature progenitor cells. HPSCs can be obtained from either the inner cell mass of a human blastocyst, termed human embryonic stem cells (hESCs), or from human somatic cells that have been reprogrammed to a pluripotent state, termed induced pluripotent stem cells (iPSCs) (**Figure 1A**).

Remarkable progress has been made over the past decade unraveling the developmental cues involved in islet cell formation. We now have robust step-wise differentiation protocols that mimic the key fate decisions for directing hPSCs into pancreatic endocrine cells using small molecules and growth factors *in vitro (* 134–139). These differentiation protocols efficiently guide hPSCs towards pancreatic endoderm cells (PDX1^+^/NKX6.1^+^) in four “stages”, followed by commitment to the pancreatic endocrine lineage (NEUROG3^+^), then insulin-secreting endocrine cells (INS^+^/NKX6.1^+^), and finally to β-like cells capable of glucose-induced insulin secretion (INS^+^/MAFA^+^/UCN3^+^) (3) (**Figure 1B**). The challenge in recent years has been understanding the final stages of human β-cell maturation so we can generate fully mature β-cells with a rapid and robust insulin secretory response to various secretagogues. Despite these limitations, we believe that hPSCs are an excellent tool for studying adverse effects of environmental contaminants both during pancreas development and in adult pancreatic endocrine cells. Indeed, a recent study by Zhou et al. in *Nature Communications* beautifully demonstrated the diverse and powerful applicability of hPSCs for high-content screening of potential β-cell toxins, exploring gene-environment interactions, and comparing toxicity in diverse cell types (140). The authors differentiated hESCs into INS^+^ cells in a 384-well plate format and screened a U.S. Environmental Protection Agency (EPA) ToxCast library of ~ 2,000 compounds for “hits” that impaired survival of INS^+^ cells (140). Using this study as an example, we highlight the numerous benefits of using hPSCs, whether hESCs or iPSCs, for exploring MDC toxicity.

### Flexibility to Model Developing or Adult Cells

HPSCs offer a flexible model to test for MDCs that impact either the early formation of β-cells during fetal development or the function and survival of adult insulin-secreting β-like cells. Depending on the research question, we propose two different workflow approaches. For developmental studies (**Figure 1B**, Workflow #1), environmental toxin(s) can be introduced to differentiating hPSCs at critical days or “stages” of differentiation. Thus, a critical aspect of this workflow is establishing hPSC differentiation protocols within the toxicology lab conducting chemical testing. The impact of toxins can be assessed by measuring key pancreatic cell markers by flow cytometry, image-based analysis, qPCR, or other techniques that are amenable to high throughput analysis. For example, the proportion of cells expressing markers of pancreatic commitment (% PDX1^+^), pancreatic endoderm (% PDX1^+^/NKX6.1^+^), and induction of the endocrine program (% NEUROG3^+^) are excellent benchmarks for early stages of differentiation. At later stages, the proportion of cells that acquire insulin (% INS^+^/NKX6.1^+^) is as an indicator of commitment to the β-cell lineage, and subsequently the proportion of INS^+^ cells co-expressing critical β-cell markers such as MAFA is an important indicator of β-cell maturity. Fluorescent reporter hPSC lines generated by genome editing – for example, NEUROG3-EGFP or INS-GFP hESCs – will be particularly useful for efficient image-based screening or high-content flow cytometry applications to identify MDCs that disrupt the formation of key pancreatic cell populations (141–144).

The workflow and endpoints for toxicity studies in adult cells could differ considerably from developmental studies (**Figure 1C**, Workflow #2). First, it is feasible for hPSC-derived β-like cells to be mass-produced in large quantities at a central location to generate a reproducible starting point for toxicology screening studies. Once hPSC-derived β-like cells are validated, they can be distributed to toxicology laboratories for testing of individual chemicals or complex chemical mixtures. This is important because it separates the need for toxicity testing capacity and stem cell differentiation expertise to be housed within the same lab. As with the progenitor cell model, there are numerous potential outcomes that could be assessed in a high-throughput screening platform, such as the expression of key β-cell markers using live-cell imaging or flow cytometry, β-cell survival as in Zhou et al. (140), and basal or glucose-induced insulin secretion. Any of the outcome measures described in the section on “Toxicity Testing in Pancreatic β-cells” could be assessed in hPSC-derived β-like cells, although not necessarily in a high-content format.

It is important to recognize that current differentiation protocols generate human β-like cells with a blunted insulin secretory response to glucose compared to primary human islets (134–139). For the purpose of identifying MDCs that cause β-cell dysfunction or apoptosis, we propose that generating fully mature human β-cells *in vitro* may not be a necessary milestone. Instead, the benefits of a large-scale source of expandable stem cells that can generate large quantities of moderately glucose-responsive insulin-secreting cells outweighs the downside of working with a slightly immature β-like cell. This has certainly proven true for toxicity studies in other cell types, such as cardiomyocytes, where differentiation protocols currently generate immature cardiomyocytes, but recapitulate sufficient features of adult cells to study adverse drug reactions in specific aspects of cardiotoxicity (145–148).

### Scalability and Reproducibility

The scalability of hPSCs is a significant advantage for high-content screening. Importantly, hPSCs share the proliferative advantage of immortalized β-cells, but subsequently lose this capacity as they differentiate into pancreatic lineage cells (139). The highly proliferative nature of hPSCs allows them to be substantially expanded before differentiation, 50–100 fold per week, particularly when grown in suspension format (149). Large batches of hPSCs can then be differentiated into a mass-produced cell product, which can be carefully validated with well-defined QA/QC protocols before being frozen down and distributed for toxicity testing (**Figure 1C**, Workflow #2). This is similar to the model proposed by the diabetes cell therapy field for mass-production of a GMP-grade cell product for transplantation (3, 149). Alternatively, more modest scale-up approaches can be established within the same lab that will perform toxicity endpoint assessments (**Figure 1B**, Workflow #1). For example, Zhou and colleagues expanded hESCs in a more traditional adherent format with Matrigel-coated plates before seeding dissociated cells into 384-well plates for pancreatic differentiation and chemical screening (140). The incredible flexibility to both scale-up hPSC production and differentiation or to miniaturize pancreatic differentiation is an important benefit of using hPSCs for toxicity studies.

### Unique Capacity for Disease Modeling

Stem cells offer remarkable capacity for disease modeling through both the natural genetic diversity of iPSCs (150) and the ability to create isogenic hPSC lines using genome editing (151). The use of hPSCs for disease modeling in diabetes has been reviewed elsewhere (152), but here, we briefly discuss the benefits of toxicity testing in human β-cells with diverse genetic backgrounds. There is much to be learned from comparing the impact of environmental contaminants on β-like cells generated using iPSCs from a spectrum of patients with different types of diabetes (T1D, T2D, maturity onset diabetes of the young (MODY), or neonatal diabetes) or known genetic risk factors for diabetes (153–160), relative to iPSCs derived from control subjects. One particularly exciting avenue to explore in the context of T1D is how environmental toxins influence immune interactions between iPSC-derived β-cells and autologous immune cells from the same donor (159). Zhou and colleagues also demonstrated the potential for using iPSCs to explore mechanisms of toxicity (140). They used 10 different iPSC lines with heterogeneous expression of a phase 2 xenobiotic metabolism enzyme, *GSTT1*, and found that pesticide-induced INS^+^ cell death was significantly higher in lines lacking *GSTT1* compared to those with at least one copy of *GSTT1 (* 140). Their results were also validated in isogenic hESC lines with *GSTT1* deletion by CRISPR-based genome editing; INS^+^ cells generated from *GSTT1^-/-^* hESCs were more susceptible to pesticide-induced cell death than INS^+^ cells from wildtype hESCs. Importantly, with the advent of CRISPR-Cas9 technology, modifying the genome of hPSCs has become broadly accessible and the number of gene-edited hPSC lines that effectively recapitulate different aspects of diabetes-related phenotypes is increasing rapidly (140, 151, 155, 161–164).

One final consideration for disease modeling is that despite being reprogrammed back to their embryonic/pluripotent state, iPSCs retain DNA methylation marks, lineage bias, and other memory of previous environmental exposures (165). For this reason, there is a strong argument for developmental models of pancreatic toxicity being limited to hESCs rather than iPSCs. On the other hand, the genetic variability of iPSCs, combined with the ability to create targeted genome-edited hPSC lines with isogenic wildtype controls, should be harnessed to explore the biological diversity of gene-environment interactions in adult β-like cells.

### Diversity of Human Cell Types

Another unique advantage of hPSCs is their ability to be directed into diverse cell types. For example, the toxicology field is already using hPSCs to test for adverse drug reactions in iPSC-derived hepatocyte-like cells to model hepatoxicity (166–168) and iPSC-derived cardiomyocytes to model cardiotoxicity (145–147, 168–172). While our Perspective focused on the application of hPSCs for toxicity testing in pancreatic lineage cells specifically, there is immense value in a more integrated approach to screen for MDCs that adversely impact different metabolic target tissues, all derived from the same hPSC source. For example, Zhou and colleagues differentiated hESCs into CD29^+^/CD73^+^ mesenchymal stem cells, CTNT^+^ cardiomyocytes, A1AT^+^ hepatocytes, and HuC/D^+^ neurons (140). They found that much like hESC-derived INS^+^ cells, HuC/D^+^ neurons were also highly susceptible to pesticide-induced cell death, suggesting that the pesticide flagged in their high-content screening could be involved in the pathogenesis of both diabetes and Parkinson’s disease. An even more complex, but intriguing application of hPSCs is the potential to develop multi-organ systems in a microfluidic device (173) or other platform containing numerous hPSC-derived metabolic tissues such as liver, adipose, and β-cells to determine how environmental contaminants influence metabolic tissue cross-talk.

## Conclusion

Despite the critical importance of pancreatic endocrine cells for maintaining metabolic homeostasis, the pancreas has not traditionally been studied as a key target tissue of chemical toxicity. Given the metabolic-disrupting nature of many environmental pollutants, we propose that islet toxicity should be considered a key toxicological endpoint. With the staggering number of poorly studied chemicals in commerce, physiologically relevant models that can be scaled up for efficient chemical screening are urgently needed. Human stem cells offer a unique solution to many of the limitations posed by other *in vitro* model systems of pancreatic endocrine cells. Most importantly, hPSCs are scalable and amenable to high-throughput screening for assessing the impact of environmental contaminants on either adult β-like cells or critical windows of pancreas development.

## Author Contributions

EM and JB conceived the review topic and wrote the manuscript. All authors contributed to the article and approved the submitted version.

## Funding

This research was supported by a Canadian Institutes of Health Research (CIHR) Project Grant (#PJT-2018-159590).

## Conflict of Interest

The authors declare that the research was conducted in the absence of any commercial or financial relationships that could be construed as a potential conflict of interest.
